# Effects of maternal and post-weaning supplementation with microbe-derived antioxidants on sow and piglet performance, oxidative status, and gut microbiota

**DOI:** 10.3389/fvets.2025.1574259

**Published:** 2025-06-11

**Authors:** Jiayong Tang, Yifan Wang, Qiang Zhou, Zhengfeng Fang, Yan Lin, Shengyu Xu, Bin Feng, Yong Zhuo, Xuemei Jiang, Hua Zhao, De Wu, Lianqiang Che

**Affiliations:** Key Laboratory for Animal Disease-resistance Nutrition of China Ministry of Education, Animal Nutrition Institute, Sichuan Agricultural University, Chengdu, Sichuan, China

**Keywords:** sows, piglets, reproductive performance, antioxidant, microorganisms

## Abstract

The antioxidants were found to improve inflammatory responses and redox status. This study investigated the effects of maternal and post-weaning supplementation with microbe-derived antioxidants (MA) on sow performance, redox status, and fecal microorganisms, as well as the growth performance, inflammatory responses and intestinal microbiota of weaned piglets. Sixty multiparous sows were randomly allocated to the control group (CON, basal diet) and the MA group (basal diet supplemented with 2.0 g MA/kg) from d 90 of gestation to d 24 of lactation, according to the parity and body condition. At weaning, a total of 80 piglets per group were selected and randomly assigned to either the basal diet or the MA-supplemented diet, with 10 pens per group and 4 piglets per pen, for a period of 21-day trial. Results showed that maternal MA supplementation increased litter size at weaning (*p* < 0.05) and the milk contents of dry matter (*p* = 0.08) and fat (*p* = 0.09), while decreasing the plasma activities of alanine aminotransferase and aspartate aminotransferase in sows on d 24 of lactation (*p* < 0.05). Moreover, maternal MA supplementation reduced plasma malondialdehyde concentration (*p* ≤ 0.01) in sows at farrowing and weaning, as well as catalase activity at weaning (*p* = 0.01), and tended to increase total antioxidant capacity at farrowing (*p* = 0.08). Additionally, the fecal contents of butyrate (*p* = 0.04) and propionate (*p* = 0.09) were higher in sows receiving the MA diet at d 24 of lactation. In post-weaning piglets, maternal MA supplementation increased average daily gain (*p* = 0.07) and average daily feed intake (*p* < 0.05) throughout the period, and increased plasma immunoglobulin G and interleukin-10 concentrations (*p* < 0.05). Additionally, either maternal or post-weaning MA supplementation positively influenced the gut microbiome of both sows and weaned piglets. In conclusion, maternal MA supplementation during late gestation and lactation increased litter size at weaning, which may be associated with the improved milk quality and redox status. Furthermore, maternal MA supplementation may enhance the growth performance of post-weaning piglets, potentially linking to the improvements in immunological parameters and gut microbiome.

## 1 Introduction

During late gestation and lactation, sows experience stress due to environmental and physiological changes, which include feeding restrictions, housing modifications, and farrowing transitions. The perinatal period is particularly critical for both dam and offspring, as it brings significant changes to maternal metabolism, redox status, and microbiota (1, 2). Physiological changes that occur in sows during farrowing, such as the production of prostaglandins, prostacyclin, and tissue damage, can lead to inflammatory reactions in sows (3). Additionally, the levels of pro-inflammatory factors during early lactation are significantly higher than those during early pregnancy or late lactation (4). Furthermore, oxidative stress is observed during late gestation and lactation, which would adversely affect milk production, reproductive efficiency, and sow longevity (5).

Weaning represents a pivotal stage for the growth and development of piglets. Weaning stress, which includes dietary changes, maternal separation, environmental alterations, and more, can lead to a series of issues in piglets, such as diarrhea, morbidity, growth check etc. (6, 7). Various feed additives, such as cysteamine, plant extracts, probiotics, and postbiotics, had been shown to alleviate weaning stress by reducing diarrhea, improving redox status and microbiota composition (8–10). Recent studies have demonstrated that maternal nutrition or supplementation with functional components (such as postbiotics) can promote improvements in both the growth performance and health of offspring (11–13).

Postbiotics, also called inactivated probiotics, are a complex mixture of inactivated bacterial cells generated through fermentation and probiotic metabolites, such as secreted proteins, peptides, enzymes, proteins, vitamins, and exopolysaccharides (EPSs), which provide overall health benefits to animal (14, 15). Studies have shown that maternal supplementation with yeast-derived postbiotics can decrease piglet mortality and the diarrhea index (16). The postbiotic consisting of heat-inactivated *Lactobacillus fermentum* and *Lactobacillus delbrueckii* improved the growth performance of nursery pigs, which was related to enhanced intestinal health and increased diversity and abundance of beneficial microbiota in pigs challenged with F18^+^ *E. coli* (17). Meanwhile, *Lactobacillus reuteri* postbiotics can also improve antioxidant function and reduce piglet mortality by regulating the gut microbiota (18).

Microbe-derived antioxidants (MA) are a mixture of fermented and inactivated *Sea buckthorn* and *Rosa roxburghii* combined with probiotics, containing numerous bioactive compounds such as isoflavones, flavone, glutathione, and polyphenols etc. (19, 20). As a type of postbiotic, studies have been reported that MA exhibits free radical scavenging ability (21), inhibits oxidative stress and inflammation (22), possesses hypolipidemic and anti-inflammatory properties (12, 23). Dietary supplementation with MA during gestation and lactation in rats can alleviate high-fat diet-induced production of hepatic nitrogen radical, increase antioxidant enzyme activity, and attenuate lipid accumulation and NLRP3 inflammasome levels (12). Considering these potential benefits, we hypothesized that MA could enhance the productive performance and health status of sows and their offspring. Therefore, this study aimed to investigate the effects of maternal and post-weaning MA supplementation on the productive performance of sows and their offspring, gut microbiota, redox status and immunology-related parameters.

## 2 Materials and methods

### 2.1 Experimental materials

Microbe-derived antioxidants was provided by Shanghai Chuangbo Ecological Engineering Co., Ltd. (Shanghai, China). Briefly, it was produced through a multi-stage complex fermentation process involving *Rosa roxburghii* and *Sea buckthorn* with *Bacillus Subtilis*, *Lactobacillus*, *Clostridium Butyricum*, and *Saccharomyces cerevisiae*. The process included extraction, concentration, inactivation, and freeze-drying. MA exhibited a fermented taste, with the main bioactive compounds being 1.37% isoflavones, 194,000 U/100 g superoxide dismutase, 886 mg/100 g glutathione, and 82.4 mg/100 g total saponins (19, 20).

### 2.2 Animals, experiment design and management

Sixty Landrace × Yorkshire sows (LY, parity 4.06 ± 0.24) at 90 days of gestation were randomly assigned to two groups based on parity and body condition, with 30 replicates for each group and one sow per replicate (*n* = 30). The control group (CON) received basal diet, whereas the treatment group (MA) received the basal diet supplemented with 2.0 g MA/kg. The nutritional requirements for the basal diet of sows during gestation and lactation were based on the National Research Council (2012) (NRC, United States), and the compositions and nutritional levels of the diets were detailed in Supplementary Table S1. The sow trial extended from d 90 of gestation to d 24 of lactation.

At weaning, a total of 80 Duroc × Landrace × Yorkshire (DLY) piglets were selected from each group based on their litter (2–3 piglets per litter) and body weight (BW, the average weight per litter). The 80 piglets from each group of sows were then randomly assigned to receive either a basal diet or a basal diet supplemented with 2.0 g MA/kg. The two factors, maternal diet and post-weaning diet, were arranged in a 2 × 2 factorial design, resulting in four treatment groups, with 10 pens for each group (*n* = 10) and 4 piglets per pen, as follows: CON. CON (basal diet for both sows and piglets), CON. MA (basal diet for sows and MA diet for piglets), MA. CON (MA diet for sows and basal diet for piglets) and MA. MA (MA diet for both sows and piglets). The basal diet was formulated according to the nutritional requirements of 5–7 kg and 7–11 kg piglets, as outlined by the NRC (2012), and the ingredients and nutrient composition were provided in Supplementary Table S1. After 3 days of pre feeding, the piglets trial lasted 21 days until 24 d post-weaning.

The feeding, management, and immunization procedures were conducted according to the farm’s standards for gestating and lactating sows and weaned piglets. During gestation, all the sows were housed in individual stalls and fed 3 kg/d of gestation-period diet twice a day (08: 00 and 15:00), with access to water *ad libitum* throughout the study. On d 110 of gestation, the sows were moved to the farrowing room and the day of parturition was defined as d 0 of lactation. The sows were no feed on the day of farrowing, 2 kg/d of diet on the following 3 days, and then feed freely twice a day (8:00, 16:00). Feed allocation and refusals were recorded daily during lactation. At farrowing, the numbers of live, stillborn, and mummified piglets, along with their birth weight, were recorded. Cross-fostering occurred within 48 h post-farrowing and the litters were adjusted to consist of 11–12 piglets within the same treatment. The numbers and weights of piglets were recorded after standardizing the litters and again at weaning. Two sows in the CON were eliminated due to fever and illness before production. Additionally, the piglets had free access to water throughout lactation but did not have access to creep feed. Weaned piglets were fed *ad libitum* four times a day (08:00, 12:00, 16:00, and 20:00). During the trial, weaned piglets had free access to water; pens were regularly cleaned, disinfected, and dewormed, with stable air circulation and temperature maintained. Fecal scores of pigs were recorded daily following observations of the individual piglet and signs of stool consistency in the pen. The calculation of fecal scoring criteria and diarrhea index was performed as previously described by our group (24). On days 0, 7, 14, and 21 of formal trial, all piglets were weighed and feed intake was recorded. The average daily gain (ADG), average daily feed intake (ADFI) and feed to gain ratio (F/G) were calculated for each pen.

### 2.3 Samples collection

Colostrum samples (15 mL) were collected from 8 sows per group (*n* = 8) before any piglets sucked, and milk samples (15 mL) were obtained from the same sows (*n* = 8) on d 10 of lactation. The milk samples were collected using 2 mL of oxytocin via the ear vein, as previously described (11). All samples were stored at −20°C for subsequent analysis.

Blood samples were collected in sodium heparin-anticoagulated vacuum tubes. Fasting blood samples (10 mL) were collected from marginal ear vein of 8 sows per group (*n* = 8) at 1 h post-farrowing and on d 24 of lactation, before the morning meal. Fasting blood samples (5 mL) were collected from the anterior vena cava of 8 piglets (one per pen, *n* = 8) per group on days 0 and 21 of formal trial. The blood was maintained at room temperature for 30 min, then centrifuged at 3,000 × g and 4°C for 10 min. The supernatant plasma was collected and stored at −20°C for later analysis.

Eight healthy sows (*n* = 8) were randomly selected from each group, and fresh fecal samples were collected immediately after defecation (one sample per pig). The samples were collected in sterile centrifuge tubes 1 h after the first piglet was born and on d 24 of lactation, placed in liquid nitrogen, and then stored at −80°C for microbial analysis. At the end of the weaned piglet trial, 8 pigs per group (one per pen, *n* = 8) were selected and anaesthetized with a lethal injection of sodium pentobarbital (200 mg/kg BW) following an overnight fast. The abdomen was then immediately opened, the jejunum was removed, emptied, and washed with normal saline. The middle section of the jejunum (1.5 cm) was collected and fixed in a 4% paraformaldehyde solution for histological analysis. Additionally, colonic chyme from the mid-region of the colon was collected in sterile centrifuge tubes, quickly placed in liquid nitrogen, and then stored at −80°C for microbial analysis.

### 2.4 Milk and plasma indicators analysis

The contents of fat, protein, urea nitrogen and lactose were determined using a multifunctional dairy analyzer (MilkoScan FT+, FOSS, Sweden). The dry matter (DM) was measured using method (930.15) of AOAC (2019) (25).

Alanine aminotransferase (ALT), aspartate aminotransferase (AST) and gamma glutamyl transferase (*γ*-GGT) in sow plasma were measured using reagent kits (CH0101201, CH0101202, CH0101204; Maccura, Chengdu, China) on an automatic biochemistry analyzer (3,100, HITACHI, Tokyo, Japan). The activities of catalase (CAT), total antioxidant capacity (T-AOC), and total superoxide dismutase (T-SOD), along with the concentrations of malondialdehyde (MDA), protein carbonyl (PCO) and inhibition and produce superoxide anion (O_2_^−^), were quantified using corresponding assay kits (No. A007-1-1, A015-1-2, A001-1-2, A003-1-2, A087-1-2 and A052-1-1; Jiancheng Bioengineering, Nanjing, China), following the manufacturer’s instructions. The concentration of protein was determined using the bicinchoninic acid (BCA) method with a BCA protein assay kit (No. A045-3-2, Jiancheng Bioengineering, Nanjing, China).

In piglet plasma, IgG, IgM and complement 3 (C3) were measured using reagent kits (CH0105306, CH0105308, CH0105309; Maccura, Chengdu, China) on an automatic biochemistry analyzer (3,100, HITACHI, Tokyo, Japan). The levels of interleukin-10 (IL-10), tumor necrosis factor (TNF-*α*) and interleukin-1β (IL-1β) were measured using ELISA kits (No. P1000, PTA00 and PLB00B; R&D Systems China Co., Ltd., Shanghai, China), following the manufacturer’s instructions.

### 2.5 Histomorphology measurements

Jejunum samples of the piglets, preserved in 4% paraformaldehyde solution, were dehydrated and infiltrated with paraffin wax. This procedure was performed as previously described by our group (24). The samples were sectioned to a thickness of 5 μm using a microtome (HistoCore MULTICUT, Leica, Wetzlar, Germany) and stained with periodic acid-Schiff. Crypt depth (CD) and villus height (VH) of each sample were measured using Image-Pro Plus 6.0 software (Media Cybernetics, Maryland, United States) with a digital trinocular microscope camera (BX43F, Olympus, Tokyo, Japan). At least 10 well-oriented villi and crypt columns were counted for each sample, and the ratio of villus height to crypt depth (VH/CD) was calculated. Simultaneously, goblet cells and the corresponding villus area were measured, and the density of goblet cells was calculated.

### 2.6 Short-chain fatty acids analysis

Sow feces were prepared using methods previously described by our group (24). Briefly, approximately 2 g of feces was mixed with ultra-pure water (1:1, w/v) and then centrifuged for 10 min at 12,000 × g and 4°C. The supernatant was mixed with 25% metaphosphoric acid and crotonic acid, incubated and recentrifuged. Subsequently, the supernatant was mixed with methanol and recentrifuged again. Finally, the supernatant was filtered through a 0.22 μm filter membrane, and the short-chain fatty acids (SCFAs) concentrations were determined using a gas chromatograph system (CP-3800, Varian, Palo Alto, United States).

### 2.7 Microbial analysis

The microflora structure in sow feces and piglet colonic chyme was analyzed using 16S rRNA amplicon sequencing technology. This procedure was performed as previously described by our group (24). Total DNA was extracted from the samples using the Fecal DNA Isolation Kit (No. D4015-01, Omega, Norcross, United States) according to the manufacturer’s instructions. DNA purity and concentration were analyzed using 1.5% agarose gel electrophoresis and a spectrophotometer (Nanodrop 2000, Thermo Fisher Scientific, Waltham, United States). The qualified DNA samples were sent to Novogene Bioinformatics Technology (Beijing, China) for sequence analysis. The V4 hypervariable regions of the 16S rRNA gene were amplified with primers 515F (5′-GTGCCAGCMGCCGCGGTAA-3′) and 806R (5′-GGACTACHVGGGTWTCTAAT-3′). Pyrosequencing of bacterial 16S rDNA was performed using the Illumina NovaSeq platform to generate 250 bp paired-end reads. The paired-end reads were merged using FLASH (V1.2.11), quality filtering was performed, and chimera sequences were removed to obtain effective tags. The effective tags were assigned to operational taxonomic units (OTUs) using the Uparse software (V7.0.1001) with 97% sequence similarity. Subsequent analysis of a-diversity and b-diversity was based on these normalized output data. Species annotation and *α*-diversity analysis were performed using QIIME software (V1.7.0). The LEfSe analysis was performed using LEfSe software (V1.0).

### 2.8 Statistical analysis

The data were normally distributed after testing homogeneity of variances and normal distribution using the Shapiro–Wilk method in SAS 9.4 (SAS Institute Inc., Cary, NC). The measurement data from sows were analyzed using the *T*-test procedure in SAS 9.4. Reproductive performance was measured in litters, while other indicators of sows were assessed in duplicate individuals. The data from weaned piglets were analyzed using the MIXED procedure in SAS 9.4 for split-plot analysis of variance and significance testing. The statistical models included the effects of sow diet MA levels (0 or 2.0 g/kg), piglet diet MA levels (0 or 2.0 g/kg), and their interaction. Piglet weaning BW was treated as a random effect, and the Tukey method was used for multiple comparisons. Except for growth performance and the diarrhea index, measured in pens, all other indicators were assessed in individual subjects. Data were presented as means ± SE, and differences were considered statistically significant at *p* < 0.05, while results with 0.05 ≤ *p* < 0.1 were defined as trends.

## 3 Results

### 3.1 Reproductive performance

As shown in Table 1, maternal MA supplementation did not significantly alter the number of piglets born alive, stillborn piglets and total born piglets, the litter weight of total born and born alive piglets, or the duration of farrowing. However, the litter size at weaning was significantly increased in the MA group compared to the CON group (*p* < 0.05).

**Table 1:** Effect of maternal MA supplementation on reproductive performance of sows and growth performance of suckling piglets^1^.

Item	CON	MA	*p*-value
Birth, piglets/litter			
Litter size (total born), no	14.46 ± 0.60	14.46 ± 0.61	0.99
Born alive, no	12.57 ± 0.79	13.14 ± 0.58	0.56
Stillborn piglets, no	1.64 ± 0.64	1.10 ± 0.24	0.44
Litter weight of total born, kg	19.17 ± 1.05	19.46 ± 0.68	0.76
Total born weight per piglet, kg	1.34 ± 0.06	1.33 ± 0.04	0.91
Litter weight of born alive, kg	17.27 ± 1.28	18.19 ± 0.68	0.51
Born alive weight per piglet, kg	1.37 ± 0.06	1.38 ± 0.03	0.80
Duration of farrowing, min	215 ± 27	186 ± 14	0.35
After cross-fostering			
Litter size by cross-fostering, no	11.75 ± 0.18	11.97 ± 0.20	0.28
Litter size at weaning, no	10.74 ± 0.20^b^	11.53 ± 0.55^a^	0.02
Litter weight by cross-fostering, kg	20.11 ± 0.64	19.95 ± 0.67	0.87
Litter weight at weaning, kg	62.30 ± 2.26	62.30 ± 2.02	0.99
Feed intake during lactation, kg/d	5.86 ± 0.17	5.79 ± 0.11	0.74

CON, control; MA, microbe-derived antioxidants. ^a,b^Mean values within a row with different letter superscripts are significantly different (*P* < 0.05). ^1^Values are means ± standard error; *n* = 28, *n* = 30 for the CON and MA groups, respectively.

### 3.2 Composition of colostrum and milk

Maternal MA supplementation tended to increase the contents of dry matter (*p* = 0.08) and fat (*p* = 0.09) in the milk compared to the CON group (Table 2).

**Table 2:** Effect of maternal MA supplementation on the composition of colostrum and milk of sows^1^.

Item	CON	MA	*P*-value
Colostrum			
Dry matter, %	25.17 ± 0.70	26.20 ± 0.55	0.26
Protein, %	15.05 ± 0.60	16.29 ± 0.60	0.11
Fat, %	3.78 ± 0.30	3.61 ± 0.18	0.64
Lactose, %	3.05 ± 0.08	2.98 ± 0.08	0.58
Urea nitrogen, mg/dL	62.58 ± 2.11	66.04 ± 2.80	0.34
Milk			
Dry matter, %	20.17 ± 0.34	22.04 ± 0.95	0.08
Protein, %	5.05 ± 0.14	5.21 ± 0.08	0.34
Fat, %	7.09 ± 0.35	8.82 ± 0.89	0.09
Lactose, %	5.93 ± 0.09	5.84 ± 0.10	0.53
Urea nitrogen, mg/dL	55.91 ± 1.07	58.87 ± 2.15	0.23

CON, control; MA, microbe-derived antioxidants. ^1^Values are means ± standard error; *n* = 8 for each group.

### 3.3 Plasma metabolites and antioxidant enzyme activities

Maternal MA supplementation significantly reduced the levels of ALT (*p* = 0.01), AST (*p* = 0.03), MDA (*p* < 0.01), and CAT activity (*p* = 0.01) in the plasma of sows at weaning, as well as the level of MDA in the plasma at farrowing (*p* = 0.01). Additionally, it tended to increase T-AOC activity in the plasma of sows at farrowing (*p* = 0.08) (Table 3).

**Table 3:** Effect of maternal MA supplementation on metabolic and redox status-related parameters in plasma of sows^1^.

Items	CON	MA	*P*-value
At parturition			
ALT, U/L	25.13 ± 1.50	25.73 ± 2.28	0.83
AST, U/L	29.52 ± 3.75	30.49 ± 5.73	0.89
γ-GGT, U/L	32.48 ± 3.95	29.17 ± 3.57	0.54
MDA, nmol/mL	2.41 ± 0.17^a^	1.89 ± 0.09^b^	0.01
O_2_^−^, U/L	205.21 ± 10.64	216.22 ± 10.64	0.47
PCO, nmol/mL	247.82 ± 7.58	247.46 ± 18.34	0.98
T-SOD, U/mL	94.97 ± 1.05	95.16 ± 1.21	0.90
T-AOC, U/mL	1.69 ± 0.03	1.78 ± 0.05	0.08
CAT, U/mL	5.52 ± 0.88	4.73 ± 0.32	0.41
At day 24 of lactation			
ALT, U/L	47.59 ± 4.31^a^	33.01 ± 2.83^b^	0.01
AST, U/L	41.73 ± 6.64^a^	25.26 ± 2.29^b^	0.03
γ-GGT, U/L	37.58 ± 4.83	29.06 ± 3.05	0.15
MDA, nmol/mL	2.76 ± 0.10^a^	2.10 ± 0.12^b^	<0.01
O_2_^−^, U/L	105.40 ± 4.45	111.08 ± 3.88	0.34
PCO, nmol/mL	472.65 ± 23.51	469.05 ± 24.38	0.91
T-SOD, U/mL	1.69 ± 0.03	1.74 ± 0.02	0.22
T-AOC, U/mL	79.43 ± 1.02	79.99 ± 1.23	0.73
CAT, U/mL	10.91 ± 1.98^a^	4.54 ± 0.99^b^	0.01

CON, control; MA, microbe-derived antioxidants; ALT, alanine aminotransferase; AST, aspartate amino transferase; γ-GGT, gamma-glutamyl transpeptidase; MDA, malondialdehyde; PCO, protein carbonyl; T-SOD, total peroxide dismutase; T-AOC, total antioxidant capacity; CAT, catalase. ^a,b^Mean values within a row with different letter superscripts are significantly different (*P* < 0.05). ^1^Values are means ± standard error, *n* = 8 for each group.

### 3.4 Short-chain fatty acids

**Figure 1:**
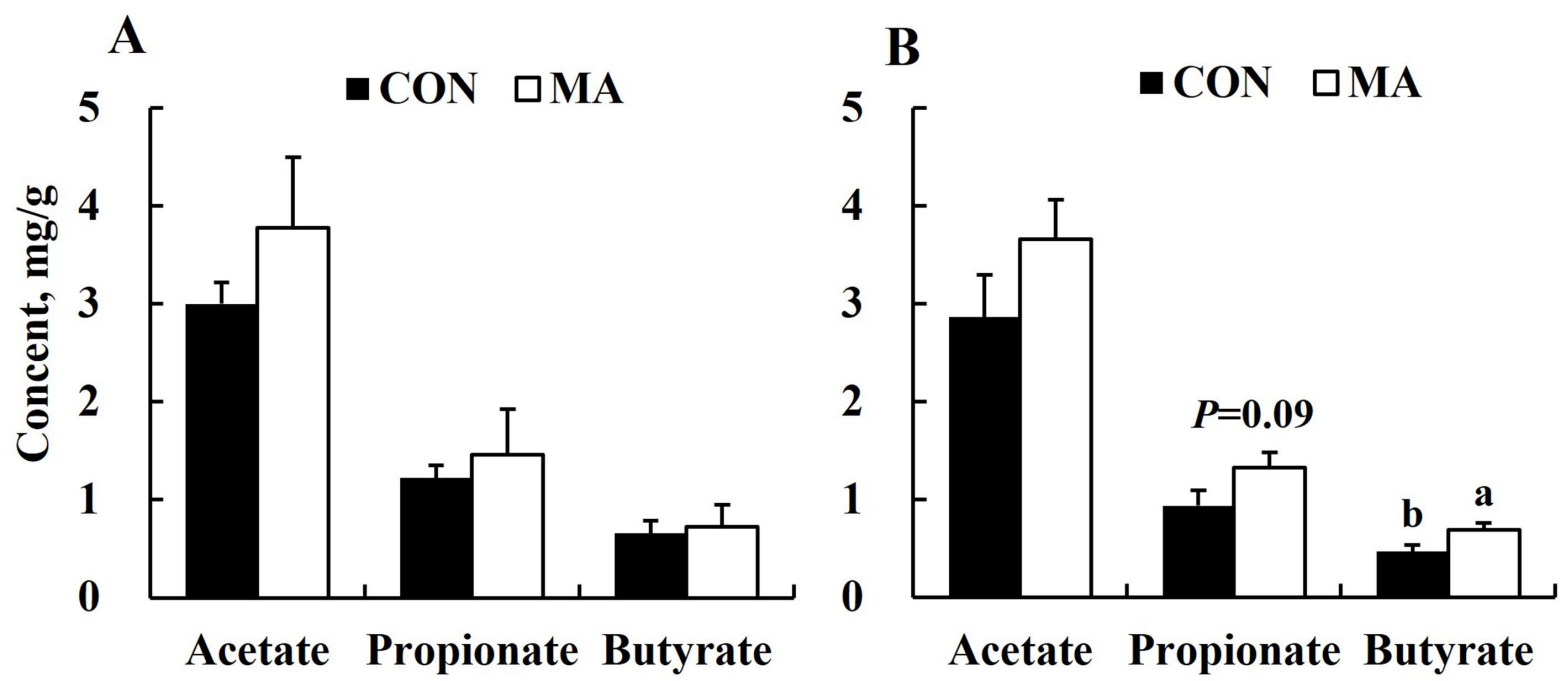
Effects of maternal MA supplementation on short-chain fatty acids of feces at farrowing and weaning. **(A)** At farrowing. **(B)** At weaning.

Maternal MA supplementation significantly increased (*p* < 0.05) butyrate levels and tended to increase (*p* = 0.09) propionate levels in sow feces at weaning (Figure 1B), but had no significant effect on acetate, propionate, or butyrate levels in feces at farrowing (Figure 1A).

### 3.5 Microbiota composition

**Figure 2:**
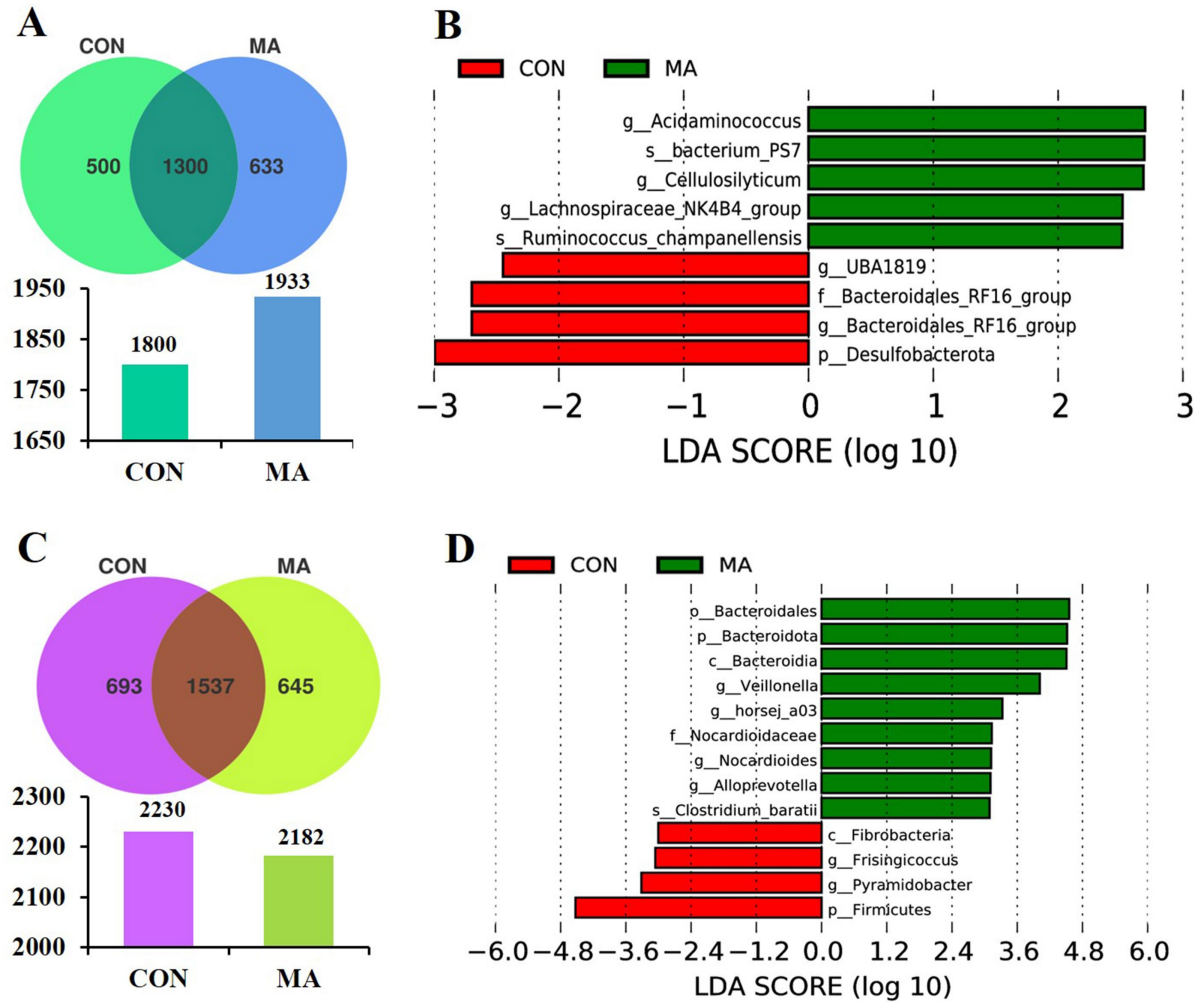
Effects of maternal MA supplementation on fecal microbial characteristics of sows at farrowing and weaning (*n* = 8). **(A)** The unique and shared OTUs in each group at farrowing. **(B)** LDA scores show the significant bacterial differences between the groups at farrowing (*p* < 0.05, LDA Score > 2.5). **(C)** The unique and shared OTUs in each group at weaning. **(D)** LDA scores show the significant bacterial differences between the groups at weaning (*p* < 0.05, LDA Score > 3.0). LDA, linear discriminant analysis; MA, microbe-derived antioxidants; OTUs, operational taxonomic units.

A total of 1800 and 1933 OTUs were assigned at farrowing (Figure 2A) and 2,230 and 2,182 OTUs were assigned at weaning (Figure 2C) in the CON and MA groups, respectively. Compared to the CON group, which had 500 unique OTUs, the MA group had more unique OTUs (633) at farrowing (Figure 2A). However, at d 24 of lactation, the MA group had 645 unique OTUs, which was 48 fewer than the 693 unique OTUs in the CON group (Figure 2C). Maternal MA supplementation had no effect on the *α*-diversity indices (Chao1, Shannon, and Simpson) at both farrowing and weaning (Supplementary Table S2).

LEfSe analysis (LDA Score > 2.5) identified 4 species (p_Desulfobacterota, f_Bacteroidales_RF16_group, *g_UBA1819*, and *g_Bacteroidales_RF16_group*) and 5 species (*g_Acidaminococcus*, *g_Lachnospiraceae_NK4B4_group 7*, *g_Cellulosilyticum*, *s_bacterium_PS* and *s_Ruminococcus_champanellensis*) enriched in the sows of CON and MA groups at farrowing, respectively (Figure 2B). In contrast, at d 24 of lactation, 9 enriched species were identified in the MA group compared to 4 species (p_Firmicutes, c_Fibrobacteria, *g_Frisingicoccus*, and *g_Pyramidobacter*) in the CON group (LDA Score > 3.0) (Figure 2D). Among the 9 species, 4 belonged to p_Bacteroidota (p_Bacteroidota, c_Bacteroidia, o_Bacteroidales, and *g_Alloprevotella*), 2 belonged to o_Propionibacteriales (f_Nocardioidaceae and *g_Nocardioides*), 2 belonged to p_Firmicutes (*g_Veillonella* and *s_Clostridium_baratii*), and *g_horsej_a03* belonged to f_Oligosphaeraceae.

### 3.6 Growth performance of piglets

As shown in Table 4, maternal MA supplementation increased the BW of piglets on d 7 (*p* = 0.05) and d 21 (*p* = 0.08) post-weaning, increased the ADG on days 1–7 (*p* < 0.05) and for the entire period (1–21 d, *p* = 0.07), also increased the ADFI on days 8–14, 15–21 and across the entire period (*p* < 0.05).

**Table 4:** Effects of maternal and post-weaning supplementation with MA on the growth performance and diarrhea index in piglets (*n* = 10).

Sow	CON		MA		*P*-value		
Piglet	CON	MA	CON	MA	Sow	Piglet	Sow×Piglet
BW, kg							
0 d	6.74 ± 0.23	6.74 ± 0.23	6.72 ± 0.22	6.73 ± 0.21	0.55	–	–
7d	6.85 ± 0.25	6.87 ± 0.25	7.00 ± 0.23	7.02 ± 0.23	0.05	0.82	0.96
14d	8.03 ± 0.28	8.06 ± 0.32	8.08 ± 0.25	8.34 ± 0.29	0.14	0.20	0.30
21 d	10.00 ± 0.40	10.18 ± 0.41	10.31 ± 0.26	10.58 ± 0.39	0.08	0.26	0.82
ADG, g/d							
1–7d	16 ± 12	19 ± 11	40 ± 10	42 ± 9	0.03	0.83	0.99
8–14 d	169 ± 16	170 ± 19	155 ± 11	189 ± 15	0.87	0.24	0.29
15–21 d	280 ± 20	303 ± 19	318 ± 16	319 ± 21	0.13	0.49	0.54
1–21 d	155 ± 10	164 ± 12	171 ± 8	183 ± 13	0.07	0.26	0.84
ADFI, g/d							
1–7 d	130 ± 8	126 ± 6	141 ± 8	129 ± 16	0.60	0.36	0.68
8–14 d	271 ± 14	275 ± 17	286 ± 8	310 ± 18	0.04	0.25	0.38
15–21 d	458 ± 29	470 ± 28	509 ± 23	532 ± 28	0.01	0.45	0.80
1–21 d	290 ± 16	290 ± 16	312 ± 12	329 ± 16	0.01	0.48	0.47
F/G, g/g							
8–14 d	1.74 ± 0.17	1.77 ± 0.07	1.92 ± 0.11	1.69 ± 0.07	0.72	0.45	0.34
15–21 d	1.65 ± 0.06	1.55 ± 0.03	1.61 ± 0.05	1.68 ± 0.04	0.23	0.69	0.36
1–21 d	1.89 ± 0.07	1.79 ± 0.05	1.84 ± 0.04	1.83 ± 0.05	0.79	0.25	0.40
Diarrhea index							
1–7 d	0.17 ± 0.03	0.17 ± 0.03	0.14 ± 0.03	0.18 ± 0.03	0.80	0.60	0.46
8–14 d	0.21 ± 0.04	0.12 ± 0.05	0.20 ± 0.05	0.19 ± 0.05	0.59	0.29	0.47
15–21 d	0.08 ± 0.03	0.11 ± 0.05	0.17 ± 0.07	0.12 ± 0.06	0.34	0.82	0.40
1–21 d	0.15 ± 0.02	0.13 ± 0.04	0.17 ± 0.05	0.16 ± 0.04	0.53	0.65	0.90

CON, control; MA, microbe-derived antioxidants; BW, body weight; ADG, average daily gain; ADFI, average daily feed intake; F/G, feed/gain.

### 3.7 Immunological and inflammatory parameters

As shown in Table 5, maternal MA supplementation tended to increase the levels of C3 (*p* = 0.07) and IL-10 (*p* = 0.06) and reduce the level of TNF-*α* (*p* = 0.07) in the plasma of piglets at weaning. Regardless of post-weaning MA supplementation, maternal MA supplementation significantly increased (*p* < 0.05) the levels of IgM and IL-10 in the plasma of piglets on d 24 post-weaning. Compared to the CON. CON group, the levels of IgM and IL-10 were markedly increased by 54.5 and 188.5% in the MA. MA group, respectively.

**Table 5:** Effects of maternal and post-weaning supplementation with MA on inflammatory parameters in plasma of piglets (*n* = 8).

Sow	CON		MA		*P*-value		
Piglet	CON	MA	CON	MA	Sow	Piglet	Sow×Piglet
0 d							
C3, g/dL	0.71 ± 0.04	–	0.83 ± 0.05	–	0.07	–	–
IgG, mg/mL	0.92 ± 0.07	–	1.02 ± 0.06	–	0.24	–	–
IgM, mg/mL	0.09 ± 0.01	–	0.11 ± 0.01	–	0.15	–	–
IL-1β, ng/mL	0.81 ± 0.15	–	0.89 ± 0.11	–	0.68	–	–
IL-10, ng/mL	2.21 ± 0.39	–	4.19 ± 0.91	–	0.06	–	–
TNF-α, pg./mL	41.15 ± 9.11	–	22.32 ± 3.54	–	0.07	–	–
21 d							
C3, g/dL	0.97 ± 0.08	1.04 ± 0.10	1.15 ± 0.06	0.97 ± 0.08	0.47	0.50	0.13
IgG, mg/mL	0.65 ± 0.05	0.66 ± 0.03	0.64 ± 0.05	0.73 ± 0.06	0.60	0.31	0.43
IgM, mg/mL	0.11 ± 0.02	0.13 ± 0.02	0.16 ± 0.01	0.17 ± 0.02	0.03	0.49	0.81
IL-1β, ng/mL	0.93 ± 0.28	0.82 ± 0.18	1.15 ± 0.21	0.81 ± 0.12	0.61	0.26	0.56
IL-10, ng/mL	4.16 ± 1.40	3.93 ± 1.44	6.76 ± 2.23	12.00 ± 3.41	0.01	0.22	0.18
TNF-α, pg./mL	49.33 ± 15.47	36.70 ± 12.51	47.20 ± 22.14	27.17 ± 8.66	0.68	0.25	0.79

CON, control; MA, microbe-derived antioxidants; C3, complement 3; IgG, immunoglobulin G; IgM, immunoglobulin M; IL-1β, interleukin-1β; IL-10, interleukin-10; TNF-α, tumor necrosis factor α.

### 3.8 Intestinal morphology

Maternal or post-weaning MA supplementation had no significant effect on the VH, CD, VH/CD ratio, or the number of goblet cells in the jejunum of the weaned piglets (Supplementary Table S3).

### 3.9 Microbial composition of colonic chyme of piglets

**Figure 3:**
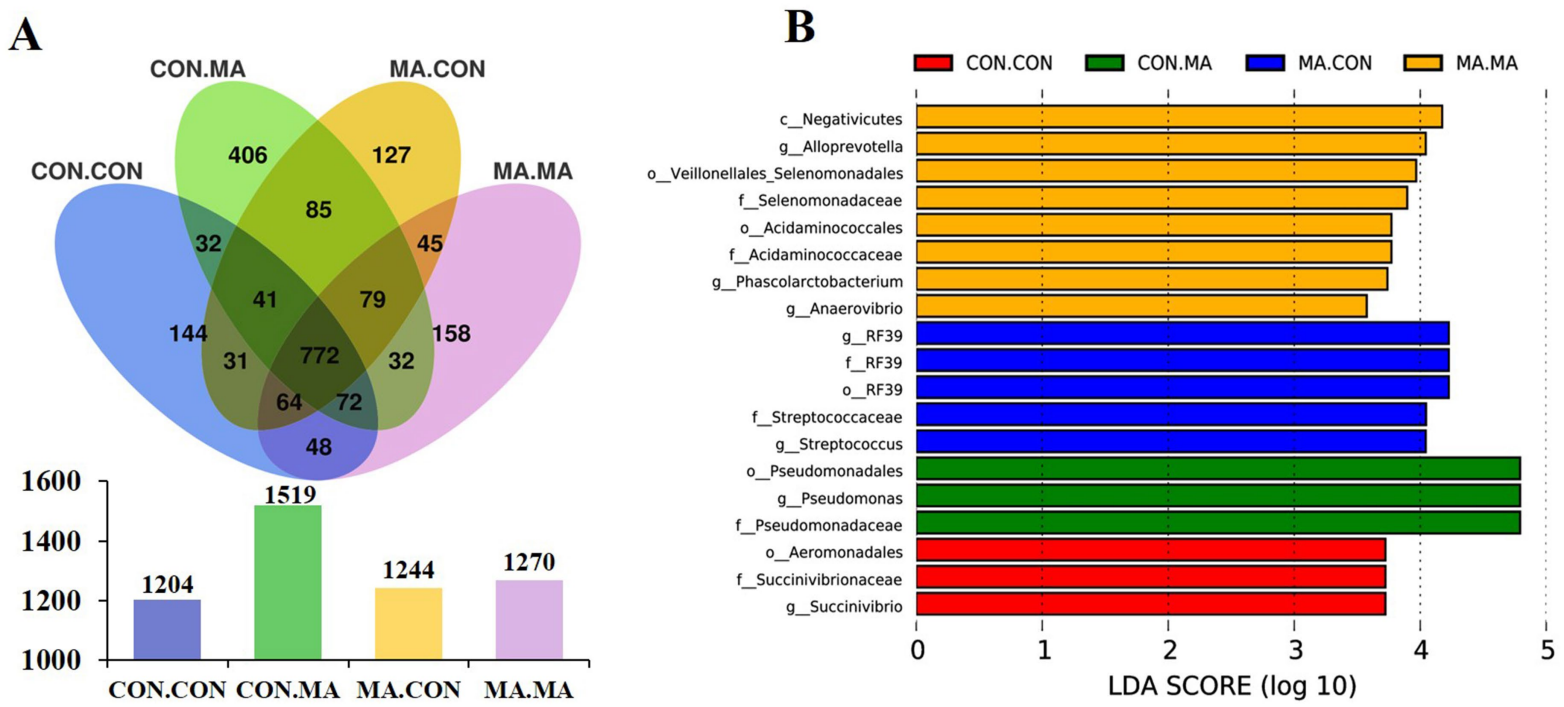
Effects of maternal and post-weaning MA supplementation on colonic chyme microbial characteristics of piglets on day 21 post-weaning (*n* = 8). **(A)** The unique and shared OTUs in each group. **(B)** LDA scores show the significant bacterial differences among the groups (*p* < 0.05, LDA Score > 3.5). LDA, linear discriminant analysis; MA, microbe-derived antioxidants; OTUs, operational taxonomic units. CON. CON, basal diet for both sows and piglets; MA. CON, 2.0 g MA/kg diet for sows and basal diet for piglets; CON. MA, basal diet for sows and 2.0 g MA/kg diet for piglets; MA. MA, 2.0 g MA/kg diet for both sows and piglets.

A total of 1,204, 1,519, 1,244, and 1,270 OTUs were assigned to the weaned offspring in the CON. CON, CON. MA, MA. CON and MA. MA groups, respectively. The four groups shared 772 OTUs, with the groups of CON. CON, CON. MA, MA. CON and MA. MA having 144, 406, 127, and 158 unique OTUs, respectively (Figure 3A). There were no significant differences in microbial *α*-diversity indices (Chao1, Shannon, and Simpson) of colonic chyme among the four groups (Supplementary Table S4).

LEfSe analysis (LDA Score > 3.5) identified 3 species (o_Aeromonadales, f_Succinivibrionaceae, and *g_Succinivibrio*), 3 species (o_Pseudomonadales, f_Pseudomonadaceae, and *g_Pseudomonas*), 5 species (o_RF39, f_RF39, f_Streptococcaceae, *g_RF39*, and *g_Streptococcus*), and 8 species enriched in the CON. CON, CON. MA, MA. CON and MA. MA groups, respectively (Figure 3B). Among the 8 species, 7 belonged to c_Negativicutes (c_Negativicutes, o_Veillonellales_Selenomonadales, o_Acidaminococcales, f_Selenomonadaceae, f_Acidaminococcaceae, *g_Anaerovibrio*, and *g_Phascolarctobacterium*), while *g_Alloprevotella* belonged to f_Prevotellaceae.

## 4 Discussion

In an intensive feeding system, hyperprolific sows experience metabolic burdens during late gestation and lactation, which may lead to oxidative stress and pro-inflammatory responses (13, 26). The oxidative stress is recognized as imbalanced redox status, an imbalance between reactive oxygen species (ROS) production and their elimination by antioxidant system, can lead to chronic inflammation (26). It has been reported that sows exhibited imbalanced redox status during late gestation and lactation, which is associated with lower levels of antioxidant components and total antioxidant capacity (5). To eliminate excessive ROS, the body produces antioxidant enzymes to maintain redox balance, such as T-SOD, CAT, GSH-Px, and nonenzymatic antioxidants. As a mixture of *Rosa roxburghii* and *Sea buckthorn* fermented by probiotics, MA contains bioactive ingredients with antioxidant properties, such as polyphenols, isoflavones, and flavones (19, 20). In this study, MA decreased MDA concentrations in the plasma of sows at farrowing and weaning, while tended to increase T-AOC at farrowing. As a byproduct of lipid peroxidation caused by ROS attacking cell membrane lipids, MDA increases with elevated oxidative stress (27). When the antioxidant status is imbalanced, a higher T-AOC indicates a stronger ability to combat oxidative stress by neutralizing excess ROS. Therefore, the higher levels of antioxidant indicators in sows receiving MA diet suggest these sows had greater antioxidant system to resist oxidative damage. Studies have shown that microbial antioxidants can enhance antioxidant capacity of sows during late pregnancy and/or lactation (11, 28). Similarly, maternal supplementation with antioxidant polyphenols (200–300 mg/kg), a class of compounds with multiple phenolic hydroxyl groups (such as flavonoids, phenolic acids, stilbenes, etc.), reduced MDA concentrations and increased the serum activities of GSH-Px and T-AOC (13).

In this study, we found that MA supplementation during late gestation and lactation significantly improved the litter size at weaning, which may be attributed to its beneficial effects on the redox status of sows. The better redox status by MA diet could be one of the factors contributing to the better milk quality, as indicated by the increasing the contents of dry matter and fat of milk from MA-supplemented sows. Similar results have been found that maternal supplementation with polyphenols, a typical antioxidant component, can increase the levels of colostrum immunoglobulins and milk composition, thereby improving pre-weaning survivability (13). Additionally, serum biochemical parameters are important indicators for evaluating the physical status of animals. ALT and AST are enzymes involved in amino acid metabolism and indicators of liver function and nutritional recovery, and their elevation in serum usually indicates liver cell damage, which is often associated with oxidative stress (29). In this study, maternal MA supplementation reduced the activities of ALT and AST in the plasma of sows at weaning, indicating a potential improvement of MA diet on liver function of sows.

The complex community of microbiota, serving as the microbial barrier of the gastrointestinal tract, plays a crucial role in the intestinal functions of pigs, including nutrient absorption, maintenance of dynamic balance of the intestinal barrier, immune regulation, and resistance to pathogen attacks (30). The composition of intestinal microbiota is influenced by multiple factors, such as diet, physiological stage, and health status. During gestation and lactation, changes in the immune system and metabolism of sows are related to the compositional dynamics of microbiota (31). Postbiotics, including enzymes, EPS, bacterial lysates, and cell wall fragments, exert beneficial effects on gut microorganisms (32, 33). In this study, maternal MA supplementation improved the composition of beneficial bacteria in the intestines of sows, such as significantly increasing the abundances of p_Bacteroidota, c_Bacteroidia, o_Bacteroidales, f_Nocardioidaceae, *g_Alloprevotella*, *g_Nocardioides*, *g_horsej_a03*, *g_Veillonella* and s_*Clostridium_baratii* at weaning, but had no effect on the *α*-diversity indices. Among these, Bacteroidota contributes to intestinal metabolism and homeostasis (34). *Bacteroidetes* is rich in carbohydrate metabolites and polysaccharide-degrading enzymes that breakdown complex nutrients into simpler forms for absorption by the animal’s intestine (35). *Alloprevotella*, a branch of *Prevotella*, can break down complex carbohydrates and dietary fibers, producing SCFAs such as acetate, propionate, and butyrate (36). In the weaned piglets, the combination of maternal and post-weaning MA supplementation significantly increased the abundances of c_Negativicutes, o_Veillonellales_Selenomonadales, o_Acidaminococcales, f_Selenomonadaceae, f_Acidaminococcaceae, *g_Anaerovibrio*, *g_Phascolarctobacterium*, and *g_Alloprevotella*. As a gram-negative genus producing SCFAs, *Phascolarctobacterium* plays a positive role in maintaining intestinal health (37). Some studies have found that a strong correlation between the enrichment of *Anaerovibrio* and the enhancement of host amino acid metabolism and nucleosides/nucleotides biosynthesis (38).

The intestinal microbiome provides an extensive repertoire of molecules and metabolites that influence host health. Among these molecules, SCFAs, derived from bacteria-dependent hydrolysis of fibers, have attracted considerable attention because of their role in host health (39). As products of the fermentation of plant polysaccharides by intestinal microbiota, SCFAs serve as important fuels for intestinal epithelial cells, enhancing gut barrier functions as well as host metabolism (40). In this study, maternal MA supplementation increased the concentrations of butyrate and propionate in the sows on d 24 of lactation. Butyrate modulates the immune response by inhibiting pro-inflammatory cytokines, while supports intestinal epithelial cell proliferation as the primary energy source (40, 41). As the primary substrate for liver gluconeogenesis, propionate influences carbohydrate metabolism, exhibits a statin-like effect by inhibiting cholesterol synthesis pathways, and demonstrates anti-inflammatory activity comparable to butyrate (42).

Weaning stress often accompanies with serious diarrhea, infection and poor growth performance due to the immature immune system during early life of pigs (43). Previous studies have shown that supplementing with inactivated (heat-killed) *Lactobacillus* can improve growth performance and feed efficiency, reduce the occurrence of diarrhea, and positively influence gut microbiota (44–46). In this study, post-weaning MA supplementation did not significantly improve diarrhea index of piglets, which is consistent with the results of intestinal morphology. However, weaned piglets from sows receiving MA diet exhibited relatively faster growth rate, which could be perhaps ascribed to the optimal redox status and maternal transfer of probiotic bacteria to their offspring. In this study, we found that the enriched *g_Alloprevotella* in sow feces at weaning was also enriched in the colonic chyme of piglets at d 24 post-weaning. Likewise, previous studies have shown that maternal supplementation with yeast-derived postbiotics positively affects the gut microbiome and immunological parameters of offspring (16, 47).

The positive effect of maternal MA supplementation on offspring piglets is also reflected by its beneficial effect on the immunity-related parameters of offspring. In this study, maternal MA supplementation tended to increase the levels of C3 and IL-10, reduce TNF-*α* at weaning, and increase IgM and IL-10 at d 24 post-weaning. It has been reported that C3 participates in the clearance of microorganisms and mediates the occurrence of inflammation (48). As an anti-inflammatory cytokine produced by inflammatory cells, IL-10 is known to prevent excessive inflammatory responses at the maternal-fetal interface (49). As a pro-inflammatory cytokine, TNF-α had been found to be decreased in MA-supplemented mouse (22). In RAW264.7 cells, MA also significantly down-regulated the level of TNF-α together with the lower accumulation of ROS (23). Similarly, several heat-killed *Lactobacillus* probiotics have shown immunomodulatory abilities by enhancing anti-inflammatory cytokines and suppressing oxidative stress in both *in vitro* and *in vivo* experimental models (50). It can be seen that the enhancement of immune function in weaned piglets should be closely related to the maternal supplementation of MA to enhance their own immune function.

## 5 Conclusion

In summary, the present results demonstrated that maternal MA supplementation during late gestation and lactation increased the number of weaned piglets, which may be associated with the improved redox status and milk composition of sows. Additionally, maternal MA supplementation increased the growth rate of post-weaning piglets, which is likely related to the improved immunological parameters and gut microbiome of both sows and weaned piglets.

## Data Availability

The original contributions presented in the study are included in the article/supplementary material, further inquiries can be directed to the corresponding author/s.
