# Supplementary material for: Effects of maternal and post-weaning supplementation with microbe-derived antioxidants on sow and piglet performance, oxidative status, and gut microbiota
Source: Front Vet Sci. 2025 Jun 11;12:1574259. doi: 10.3389/fvets.2025.1574259 (PMC12188611; doi:10.3389/fvets.2025.1574259)
Supplement: Supplementary file 1 [file Supplementary_file_1.docx]

**Effects of maternal and post-weaning supplementation with microbe-derived antioxidants** **on** **sow and piglet performance,** **oxidative status and gut microbiota**

**Supplementary Table S1** Ingredients and nutrient composition of basal diets for sows in late gestation and lactation, and offspring weaned piglets

| Items | Gestation | Lactation | Piglets |
| --- | --- | --- | --- |
| Ingredients, % | | | |
| Corn, 7.8% CP | 59.39 | 59.26 | 25.23 |
| Expanded corn | - | - | 25.00 |
| Soybean meal, 44% CP | 12.50 | 18.00 | 7.00 |
| Expanded soybean, 35% CP | - | 8.00 | 5.00 |
| Fish meal, 63% CP | 1.00 | 2.00 | 4.00 |
| Soy protein concentrate | - | - | 5.00 |
| Low protein whey powder, 3.0% CP | - | - | 10.00 |
| Whole milk powder | - | - | 10.00 |
| Wheat bran | 10.00 | 4.00 | - |
| Sugar beet meal granulate | 12.00 | 4.00 | - |
| Soy oil | 2.50 | 2.00 | 1.00 |
| Glucose | - | - | 2.00 |
| Sucrose | - | - | 2.00 |
| Choline chloride, 50% | 0.20 | 0.20 | 0.16 |
| Limestone | 1.20 | 1.12 | 0.86 |
| CaHPO_3_ | 0.98 | 0.76 | 0.28 |
| NaCl | 0.40 | 0.40 | 0.40 |
| L-Lys HCl, 98% | - | 0.08 | 0.57 |
| DL-Methionine, 99% | - | - | 0.23 |
| L-Threonine, 99% | - | - | 0.23 |
| L-Tryptophan, 99% | - | - | 0.09 |
| Zinc oxide, 75% | - | - | 0.20 |
| Acidifier | - | - | 0.50 |
| Vitamin | 0.03^1^ | 0.03^1^ | 0.05^2^ |
| Mineral premix | 0.15^3^ | 0.15^3^ | 0.20^4^ |
| Total | 100.00 | 100.00 | 100.00 |
| Nutrient composition (Calculated values, %) | | | |
| Digestible energy, MJ/kg | 14.18 | 13.97 | 15.23 |
| Crude protein, % | 13.03 | 17.69 | 19.44 |
| Crude fiber, % | 5.24 | 2.79 | - |
| Neutral detergent fiber, % | 16.31 | 9.50 | - |
| Calcium, % | 0.75 | 0.75 | 0.83 |
| Available phosphorus, % | 0.35 | 0.35 | 0.43 |
| SID Lysine, % | 0.54 | 0.88 | 1.43 |
| SID Methionine, % | 0.18 | 0.25 | 0.54 |
| SID Methionine + Cysteine, % | 0.36 | 0.48 | 0.78 |
| SID Threonine, % | 0.39 | 0.55 | 0.84 |
| SID Tryptophan, % | 0.12 | 0.17 | 0.28 |

^1^ The vitamin provided the following per kg of complete diet: Vitamin A, 4000 IU; Vitamin D_3_, 800 IU; Vitamin E, 44 IU; Vitamin K_3_, 0.50 mg; Vitamin B_1_, 1.0 mg; Vitamin B_2_, 3.75 mg; Vitamin B_6_, 1.0 mg; Vitamin B_12_, 15.0 mg; Nicotinamide, 10.0 mg; D-pantothenic acid, 12.0 mg; Folic acid, 1.3 mg; D-biotin, 0.20 mg.

^2^ The vitamin provided the following per kg of complete diet: Vitamin A, 15000 IU; Vitamin D_3_, 5000 IU; Vitamin E, 40 IU; Vitamin K_3_, 5.0 mg; Vitamin B_1_, 5.0 mg; Vitamin B_2_, 12.5 mg; Vitamin B_6_, 6.0 mg; Vitamin B_12_, 0.6 mg; Nicotinamide, 50 mg; D-pantothenic acid, 25.0 mg; Folic acid, 2.5 mg; D-biotin, 0.25 mg.

^3^ The mineral premix provided the following per kg of complete diet: Fe (FeSO_4_·7H_2_O), 80 mg; Cu (CuSO_4_·5H_2_O), 20.0 mg; Zn (ZnSO_4_·H_2_O), 100 mg; Mn (MnSO_4_·H_2_O), 25.0 mg; I (KI), 0.14 mg; Se (Na_2_SeO_3_) 0.15 mg.

^4^ The mineral premix provided the following per kg of complete diet: Fe (FeSO_4_·7H_2_O), 100 mg; Cu (CuSO_4_·5H_2_O), 6.0 mg; Zn (ZnSO_4_·H_2_O), 100 mg; Mn (MnSO_4_·H_2_O), 4.0 mg; I (KI), 0.14 mg; Se (Na_2_SeO_3_) 0.3 mg.

**Supplementary Table S2** Effect of maternal supplementation with microbe-derived antioxidants on fecal microbial alpha diversity of sows during farrowing and weaning

| Item | CON | MA | *P*-value |
| --- | --- | --- | --- |
| At farrowing | | | |
| Chao1 | 861.52±19.43 | 872.93±19.78 | 0.69 |
| Shannon | 7.66±0.14 | 7.80±0.07 | 0.44 |
| Simpson | 0.985±0.003 | 0.987±0.002 | 0.62 |
| At weaning | | | |
| Chao1 | 879.50±24.92 | 908.48±35.54 | 0.51 |
| Shannon | 7.59±0.33 | 7.77±0.11 | 0.71 |
| Simpson | 0.967±0.023 | 0.988±0.002 | 0.52 |

CON, control; MA, microbe-derived antioxidants.

**Supplementary Table S3** Effect of maternal and post-weaning supplementation with microbe-derived antioxidants on jejunum morphology in piglets (*n* = 8)

| Sow | CON | |  | MA | |  | *P*-value | | |
| --- | --- | --- | --- | --- | --- | --- | --- | --- | --- |
| Piglet | CON | MA |  | CON | MA |  | Sow | Piglet | Sow×Piglet |
| VH, μm | 417.48±27.26 | 442.72±35.99 |  | 455.70±55.36 | 489.76±38.15 |  | 0.27 | 0.44 | 0.90 |
| CD, μm | 174.93±14.52 | 183.00±21.57 |  | 190.13±21.45 | 175.70±7.74 |  | 0.82 | 0.85 | 0.52 |
| VH/CD | 2.51±0.29 | 2.58±0.25 |  | 2.63±0.44 | 2.81±0.22 |  | 0.56 | 0.68 | 0.86 |
| Goblet cell, 10^-6^/μm^2^ | 15.31±1.53 | 17.75±2.72 |  | 17.81±1.64 | 16.31±1.85 |  | 0.79 | 0.81 | 0.33 |

CON, control; MA, microbe-derived antioxidants; VH, villus height; CD, crypt depth; VH/CD, villus height / crypt depth.

**Supplementary Table S4** Effect of maternal and post-weaning supplementation with microbe-derived antioxidants on colon chyme microbial alpha diversity in piglets (*n* = 8)

| Sow | CON | |  | MA | |  | *P*-value | | |
| --- | --- | --- | --- | --- | --- | --- | --- | --- | --- |
| Piglet | CON | MA |  | CON | MA |  | Sow | Piglet | Sow×Piglet |
| Chao1 | 606.35±77.95 | 650.24±57.23 |  | 632.16±64.89 | 631.37±64.64 |  | 0.90 | 0.44 | 0.42 |
| Shannon | 7.24±0.27 | 6.97±0.37 |  | 7.11±0.37 | 7.3±0.28 |  | 0.58 | 0.67 | 0.13 |
| Simpson | 0.98±0.004 | 0.97±0.01 |  | 0.98±0.01 | 0.98±0.01 |  | 0.36 | 0.32 | 0.27 |

CON, control; MA, microbe-derived antioxidants.
